# No Guts, No Loss: Toward the Ideal Treatment for Obesity in the Twenty-First Century

**DOI:** 10.3389/fendo.2018.00442

**Published:** 2018-08-15

**Authors:** David C. D. Hope, Tricia M. M. Tan, Stephen R. Bloom

**Affiliations:** Division of Diabetes, Endocrinology and Metabolism, Imperial College London, London, United Kingdom

**Keywords:** gut hormones, obesity, GLP-1, Oxyntomodulin, glucagon, gastric bypass surgery, diabetes mellitus

## Abstract

Over the last century, our knowledge of the processes which control appetite and weight regulation has developed significantly. The understanding of where gut hormones fit into the control of energy homeostasis in addition to the rapid advancement of pharmacotherapeutics has paved the way for the development of novel gut hormone analogs to target weight loss. Currently, bariatric surgery remains the most efficacious treatment for obesity. The emergence of gut hormone analogs may provide a useful non-surgical addition to the armamentarium in treating obesity. Simply targeting single gut hormone pathways may be insufficiently efficacious, and combination/multiple-agonist approaches may be necessary to obtain the results required for clear clinical impact.

Obesity is the accumulation of excessive fat that may impair health. It is the driver for several serious non-communicable diseases, including type 2 diabetes, cardiovascular disease, hypertension, dyslipidaemia, cancer, obstructive sleep apnoea and musculoskeletal problems. Aetiologically, obesity derives from the interaction of multiple genetic factors that bias metabolism toward the accumulation of fat, combined with environmental factors including the supply of easily available energy-dense food and reduced opportunities for physical activity. The prevalence of obesity is increasing year on year. Global estimates for obesity prevalence have tripled from 4.3% (1975) to 13.2% (2016) (1).

## Current options for obesity

Lifestyle modifications remain the first line treatment for obesity, with evidence from large studies demonstrating only a modest benefit in outcomes. For example, meta-analysis of studies with a minimum of 1 year follow-up demonstrate 5–9% short term weight loss and 3–6% long term weight loss (2). It is possible to lose much more weight with incentivized high-intensity lifestyle changes, however the weight loss is often not sustainable due to counter-regulatory changes after such weight loss. This was highlighted by the long-term follow up of participants who participated in a weight loss competition, losing an average of 58.3 ± 24.9 kg at the end of the competition (3). Participants subsequently exhibited significant metabolic adaptations to counteract weight loss efforts, in particular a sustained reduction in energy expenditure up to 6 years following initial weight loss. This led to weight regain despite continued lifestyle changes. Strikingly, this reduction in energy expenditure persisted despite weight regain. This is a clear (if somewhat extreme) example of how the body defends itself against weight loss. Current lifestyle recommendations are not a sufficiently effective and/or durable strategy for weight loss and patients require other treatment options.

The choices for the pharmacological treatment of obesity have until recently been limited to orlistat, a pancreatic lipase inhibitor preventing fat absorption within the gut. Average weight loss is around 3% over 1 year, but patient perseverance with treatment is limited due to undesirable gastrointestinal side effects (4). Treatments such as sibutramine and rimonabant have been withdrawn due to psychiatric and cardiovascular side effects respectively (5, 6). Lorcaserin (Belviq®) and phentermine/topiramate (Qsymia®) are both licensed in the US but are not currently licensed in Europe. Bupropion/naltrexone has been approved in the US under the trade name Contrave® and was more recently approved in the European Union in 2015 under the trade name Mysimba®. Overall, these pharmacological treatments are characterized by relatively weak efficacies (5–10% weight loss) and potential side effects, including the possible exacerbation of anxiety or depression, which are common co-morbidities in patients with obesity.

Bariatric treatment of obesity is established as the most effective method of obtaining sustained weight loss. A recent long-term (12-year follow up) study of patients with severe obesity undergoing Roux-en-Y gastric bypass, showed an average weight loss from baseline of 45 kg at 2 years, 36.3 kg at 6 years, and 35 kg at 12 years (7). The data from the Swedish Obesity Study shows that bariatric surgery improves mortality, and reduces the rate of cardiovascular events, cancer and diabetes (8). Long-term follow-up data also show that for obese patients with diabetes, bariatric surgery is more effective than medical therapy alone in improving glycaemic control, lipid profile and quality of life, with up to 3 in 10 patients obtaining a long-term remission, defined as a HbA1c of ≤6.5% without anti-diabetic medication (9). Furthermore, mortality rates associated with bariatric surgery are low, similar to a laparoscopic cholecystectomy operation. Despite the highly effective outcomes with bariatric surgery, there are associated post-surgical complications such as post-prandial hypoglycaemia, nutritional deficiencies, and psychosocial issues which may cause long-term morbidity and should not be overlooked (10). In addition, surgery does require considerable resources in terms of specialist surgeons, facilities and follow-up; these limit the deliverability of bariatric surgery to all patients with obesity. Lastly, surgery is a “one size” solution where patients obtain varying levels of weight loss ranging from the inadequate to the excessive from the same procedure, i.e., “one size does not fit all.”

**Figure 1:**
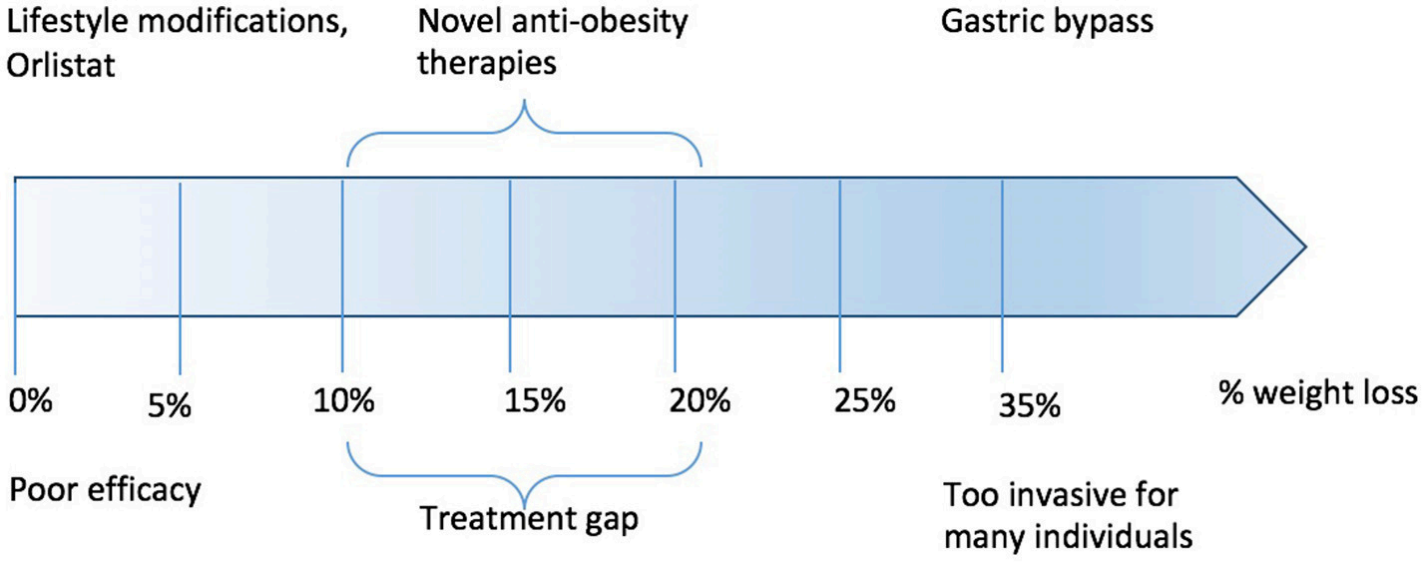
Treatment gap in obesity therapy.

Over the last 20 years, a gap has emerged between relatively ineffective pharmacological therapy and effective (but non-titratable and non-reversible) surgical treatments (Figure 1). Within this gap, there is a need for pharmacotherapies capable of inducing significant weight loss, sustaining weight loss and which are proven in reducing comorbidities such as the risk of developing overt diabetes and cardiovascular disease. In the following review, we examine the state-of-the art in gut hormone research, and the current considerable efforts to turn these into practical and safe therapies for obesity.

## Appetite and energy homeostasis

**Figure 2:**
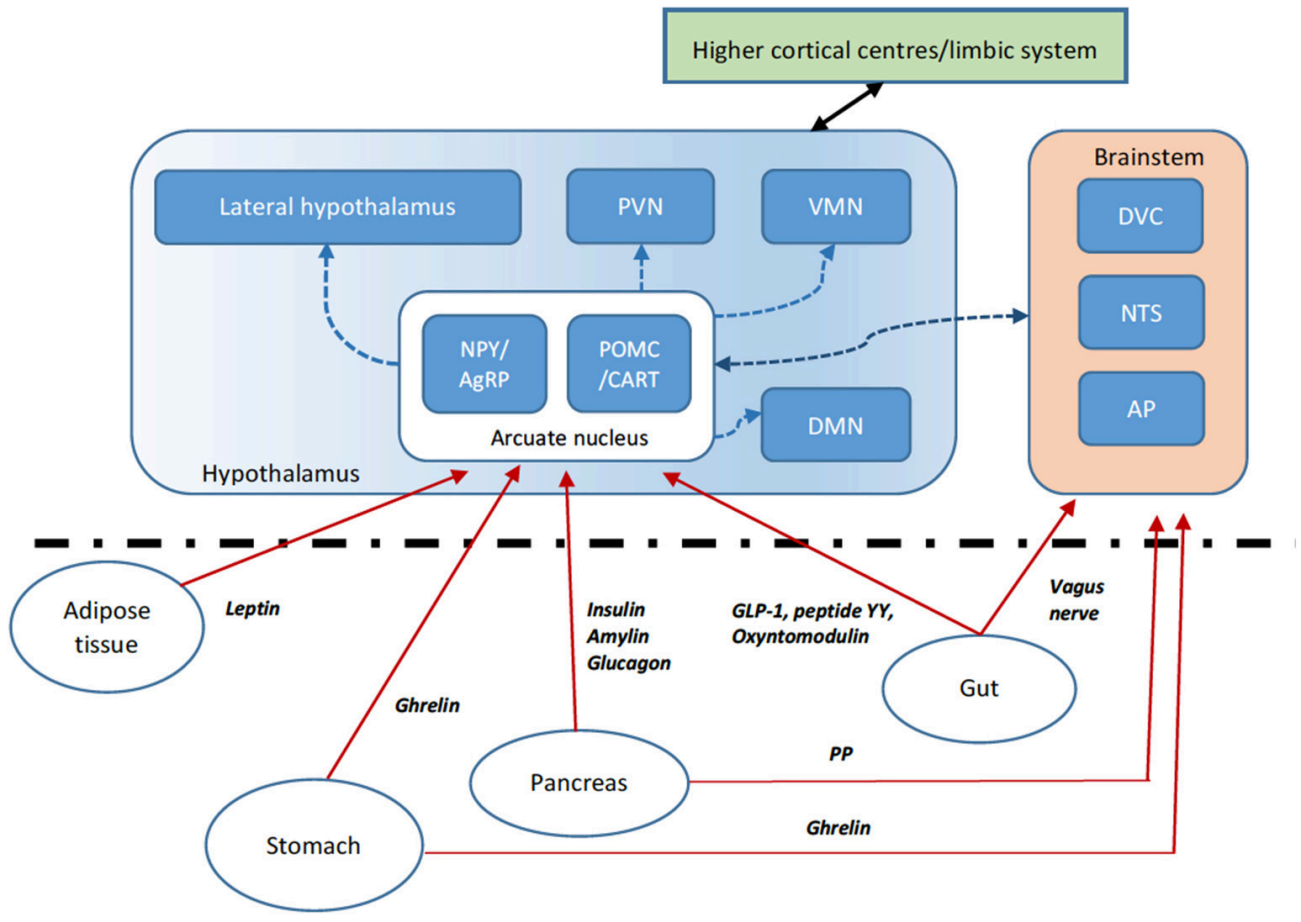
Gut brain axis schematic. The hypothalamus integrates anorexigenic and orexigenic signals. Peripheral signals such as gut peptides, leptin from adipose tissue and pancreatic signals are able to cross the blood-brain barrier at the median eminence (a circumventricular organ) or activate their cognate receptors at other circumventricular organs such as the area postrema (AP). These signals relay to the hypothalamic arcuate nucleus (ARC) via direct activation or indirectly via brainstem regions including the dorsal vagal complex (DVC) and nucleus tractus solitarus (NTS). Within the ARC, two key populations of neurons include those that express the orexigenic neuropeptide Y (NPY) and agouti-related peptide (AgRP—an inverse agonist of the melanocortin receptor MC4R) and those expressing the anorexigenic pro-opiomelanocortin (POMC), processed to the MC4R agonist alpha-MSH, and cocaine- and amphetamine regulated transcript (CART). The ARC signals to second-order neurons in various hypothalamic nuclei including the paraventricular nucleus (PVN), dorsomedial nucleus (DMN), ventromedial nucleus (VMN) and lateral hypothalamic area (LHA). Second-order neurons express anorexigenic and orexigenic neuropeptides further modulating appetite and energy homeostasis. Vagal afferents also signal directly to brainstem regions. Higher central nervous system centers including the mesolimbic pathways also signal to the hypothalamic nuclei.

The long-term control of body weight is tightly regulated through various homeostatic physiological processes to allow the body to conserve energy. Traditionally, food consumption is thought of in terms of short and long-term signals. In the acute setting, food consumption is regulated by sensory stimuli, gut mechanoreceptors, nutrient concentrations in the plasma, and changes in gut hormones secreted by specialized enteroendocrine cells. Longer term adiposity signals, for example, leptin and insulin, also influence feeding behavior. These homeostatic mechanisms rely on gut-neuronal circuits or the “gut-brain axis” allowing signaling between the periphery and central nervous system to coordinate systemic changes in our physiology (Figure 2). Both peripheral circulating peptides and vagal afferents are important in the signaling pathway controlling appetite. The main central regions where “anorexic” or “orexigenic” signals converge include brainstem regions including the dorsal vagal complex (DVC) in the medulla and the hypothalamic nuclei, most importantly the arcuate nucleus. Of the neuronal cell populations within the arcuate nucleus, two leptin-sensitive neuronal cell subtypes are characterized by the expression of specific weight regulating neuropeptides. The first contains Neuropeptide Y (NPY) and Agouti-related peptide (AgRP). The second contains alpha-melanocyte stimulating hormone (alpha-MSH) and cocaine-and amphetamine regulated transcript (CART). Activation of the orexigenic NPY/AgRP neurons leads to food intake, partly by NPY activation of neuropeptide Y1R receptors and partly by AgRP inverse agonism of the MC4R receptor. On the other hand, activation of the anorexigenic alpha-MSH/CART neurons inhibits food intake via activation of MC4R by alpha-MSH. Both pathways operate exclusively and receive signals via the nucleus of the solitary tract (NTS) and the dorsal vagal complex (DVC) of the brainstem, relaying important afferent vagal signals from peripheral gut hormone receptors, for example in the vagal innervation of the hepatic portal vein. The circumventricular organs, such as the median eminence, subfornical organs and area postrema, act as points that allow access for peripheral peptides through the blood-brain barrier or may themselves have receptors for the gut hormones, transducing these peripheral signals into the CNS (e.g., area postrema to the NTS). In addition to homeostatic mechanisms, food intake is controlled by regions of the brain regulating hedonistic pathways, circadian rhythms and cultural/learned experiences and therefore the hypothalamic nuclei also receive important signals from the cortex and mesolimbic system.

## Gut hormones

The impact of gut hormones on the regulation of appetite was discovered early on when N.F. Maclagan in 1937 investigated the effect of a crude preparation of intestinal mucosa, called enterogastrone which was administered to rabbits at a dose of 2–3 mg/kg (11). He found a transient reduction in food intake in the rabbits and reported this extract was not active by mouth. This observation is now understood to be due to the rapid degradation of gut peptides in the stomach, a process which to this day limits the widespread use of gut hormone therapy in obesity pharmacotherapy. Since Maclagan's early discovery, several gut hormones have been isolated and described (Table 1).

**Table 1:** Gastrointestinal hormones regulating food intake.

**Gastrointestinal hormone**	**Peptide family**	**Site of release**	**Stimulated by**	**Receptor(s)**	**Role in weight regulation**
Cholecystokinin (CCK)	Gastrin/CCK	Intestinal I-cells	Fat and protein rich meals	CCK1R/CCK2R	Increased satiety.
Glucagon-like peptide-1 (GLP-1)	Preproglucagon	Intestinal L-cells	Macronutrient intake	GLP-1R	Increased satiety, glucose stimulated insulin secretion, reduced gastric emptying.
Oxyntomodulin	Preproglucagon	Intestinal L-cells	Macronutrient intake	GLP-1R/GCGR	Increased satiety, glucose stimulated insulin secretion, increased energy expenditure.
Glucagon	Preproglucagon	Pancreatic alpha cells	Protein intake, stress, fasting	GCGR	Increased satiety, lipolysis, gluconeogenesis, glycogenolysis.
Peptide tyrosine tyrosine (PYY)	PP-fold	Intestinal L-cells	Macronutrient intake	Neuropeptide Y2R (PYY_3−36_). Y1R and Y5R (PYY_1−36_)	Increased satiety.
Pancreatic Polypeptide (PP)	PP-fold	Pancreatic PP cells	Macronutrient intake	Neuropeptide Y4R	Increased satiety.
Amylin	–	Pancreatic beta cells	Macronutrient intake	AMY1a, AMY2a and AMY3a	Increased satiety, reduced gastric emptying, modified food reward.
Ghrelin	Ghrelin	Stomach X/A-like cells	Fasting	GHSR	Increased food intake.

## CCK—the first “satiety signal”

The first isolated gut hormone to be investigated was cholecystokinin (CCK), a peptide released by the neuroendocrine I-cells in the duodenum and jejunum in response to fat and protein rich meals (12). The “enterogastrone” preparation used by Maclagan in his experiments contained CCK (13). Gerard Smith and colleagues showed that intraperitoneal injection of CCK into fasted rats inhibited food intake by 50% (14). To further understand the physiological role of CCK, the feeding behavior of rats with chronic gastric fistulas were studied. In a series of these experiments, CCK was proposed as the first “satiety signal” (15). CCK, in addition to inhibition of food intake, delays gastric emptying, stimulates pancreatic enzyme secretion and gall bladder contraction. The finding that CCK is a satiety signal led to research into the peptide's potential as an obesity therapy. However, CCK receptor agonists such as loxiglumide did not apparently show any anorectic activity in human volunteers and therefore their development has been abandoned (16).

## GLP-1 and its analogues as current clinical treatments for diabetes and obesity

**Figure 3:**
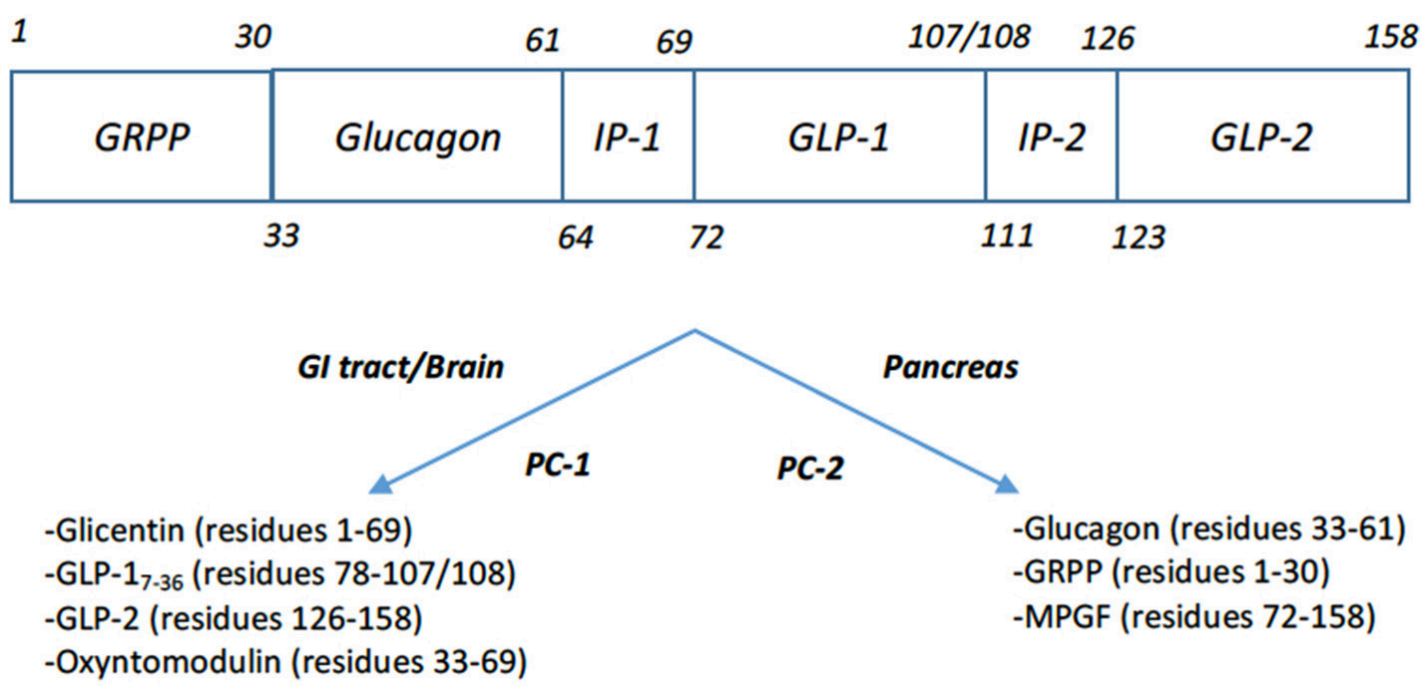
Post-translational products of Preproglucagon (17). Tissue specific processing by proprotein convertase 1 (PC-1) in the intestine and brain of proglucagon leads to formation of glicentin, GLP-1, GLP-2, oxyntomodulin. Note that GLP-1's C-terminal residue 108 is processed to the C-terminal amide. Processing of proglucagon by proprotein convertase 2 (PC-2) in the pancreas leads to the formation of glucagon, glicentin-related polypeptide (GRPP), and major proglucagon fragment (MPGF).

The most well-known therapeutic gut hormone is GLP-1 which has been developed into analogs for the treatment of type 2 diabetes mellitus over the past 20 years. GLP-1 is released from the neuroendocrine L-cells of the gut. It is the product of post-translational processing of preproglucagon, by prohormone convertase 1 (PC-1) and the peptidyl-glycine alpha-amidating monooxygenase (PHM) which adds the C-terminal amide (Figure 3). A variety of GLP-1 variants are secreted *in vivo* including the inactive forms GLP-1_1−37_ and GLP-1_1−36_NH_2_, in addition to the active forms, GLP-1_7−37_ and GLP-1_7−36_NH_2_ (18). GLP-1's role in mediating the “incretin” effect, whereby insulin secretion is enhanced when enteral glucose is given in comparison to parenteral glucose was observed early on. Wolfgang Schmidt and colleagues first postulated that GLP-1 stimulated insulin secretion and demonstrated that GLP_1−36_NH_2_ was able to stimulate glucose-dependent insulin release from isolated rat pancreatic islets, albeit at high concentrations (19). Not long after this, GLP-1_7−36_NH_2_ was shown to be a much more potent incretin in humans (20). The interest in this peptide as an obesity therapy followed *in vivo* experiments whereby GLP-1_7−36_NH_2_ was administered intracerebroventricularly (ICV) in rats, leading to a dose-dependent reduction in food intake (21). Following on from this, continuous subcutaneous infusion of GLP-1_7−36_NH_2_ in both lean and obese humans was shown to cause an increase in satiety and reduction of food intake (22, 23). In contrast to CCK, GLP-1_7−36_NH_2_ retained its anorectic effects during continuous infusion and when administered at a dose of 4.8 pmol/kg for 6 weeks led to an average weight loss of 2% (24).

One of the major breakthroughs in this area came when Buckley and Lundquist described the degradation of GLP-1 by cleavage of the N-terminal 2 residues from the peptide (25). It was later found that dipeptidyl peptidase-IV (DPP4) was responsible for this inactivation (26). Around the same time, the GLP-1R agonist exendin-4/exenatide was isolated from the saliva of the Gila monster lizard *(Heloderma suspectum*) by Eng et al. (27). This was developed into the first marketed GLP-1 analog (Byetta®) due to its resistance to DPP4 degradation, extending its half-life and making it practicable as a treatment. Since these discoveries, several GLP-1 analogs have been developed with different structural modifications to implement DPP4 resistance and prolong their half-lives. These GLP-1 analogs are mostly licensed for the treatment of type 2 diabetes, including liraglutide (Victoza®), exenatide (Byetta®), and lixisenatide (Lyxumia®). Longer acting preparations are available and include exenatide LAR (Bydureon®), albiglutide (Tanzeum®), semaglutide (Ozempic®), and dulaglutide (Trulicity®).

The advantage of the GLP-1 analogs over other types of diabetes treatment is the associated weight loss. For example, exenatide and its longer acting preparation exenatide LAR (encapsulated in poly(lactic-co-glycolic) microspheres), lead to mean weight reductions of 3.7 and 3.6 kg, respectively, over 30 weeks in a comparative study (28). When given via an annually renewed osmotic mini-pump, exenatide can reduce weight by 2.4–4.2 kg in obese type 2 diabetic patients (29). Liraglutide has a longer half-life (13 h) than exenatide, owing to the linkage of a palmitate fatty acid group by a glutamic acid spacer at position 26, therefore increasing binding to circulating albumin. Licensed doses used in clinical practice range from 0.6 to 1.8 mg daily. Liraglutide 1.8 mg has been shown to have a similar effect on weight loss to exenatide, with weight loss of 3.24 kg over 26 weeks (30). The weekly analog semaglutide, at doses of 0.5 or 1 mg weekly, has also been shown to cause mean weight reductions of 3.5-6.1 kg in trials in obese type 2 diabetics (31).

GLP-1 analogs have also been investigated as a treatment for obesity in non-diabetic patients. Astrup et al. initially showed that liraglutide at doses up to 3 mg daily reduced weight by 7.2 kg on average in a double-blind randomized placebo controlled 20-week proof of concept trial, however there was a dose-related nausea as the main side-effect (32). This proof of concept led to a 56-week randomized placebo-controlled trial in 3731 patients, where it was shown that 3 mg liraglutide led to a reduction in body weight by an average of 8.4 kg, versus the placebo group who lost an average of 2.6 kg (33). As a result, liraglutide 3 mg is now licensed as a treatment in non-diabetic obese patients (Saxenda®). Semaglutide has also been tested in non-diabetic obese subjects in a small double-blinded randomized cross-over study (at a dose of 1.0 mg weekly) and shown to reduce weight on average by 5 kg over 12 weeks in comparison with a 1 kg weight gain in the placebo arm (34). In a Phase II dose-ranging study in non-diabetic obese subjects, semaglutide was given at doses of up to 0.4 mg daily for 52 weeks and compared to liraglutide 3 mg daily: it was shown to reduce weight on average by up to 13.8% at the highest dose of 0.4 mg, compared to 7.8% with liraglutide (35).

Despite the modest weight loss effects described with the GLP-1 analogs, there are a significant proportion of patients who do not respond adequately to GLP-1 analogs in terms of weight loss: the SCALE study showed, for example, that 37% of patients assigned to liraglutide 3 mg either lost <5% baseline weight or even gained weight, in other words they were non-responders (33). Even with semaglutide 0.4 mg daily, the non-response rate was 17% (35). Another issue with GLP-1 analog therapy for obesity are the common gastrointestinal side effects (nausea, vomiting, diarrhea, constipation) which occur in up to 40% of patients, thus limiting the maximal doses that can be given.

## The PP-fold anorectic gut hormones: peptide YY, pancreatic polypeptide

Peptide tyrosine tyrosine (PYY) is a peptide co-secreted with GLP-1 from the neuroendocrine L-cells of the intestine and is a member of the pancreatic polypeptide (PP) family of proteins. The PP family of proteins contains pancreatic polypeptide (PP), PYY, and the neurotransmitter neuropeptide Y (NPY), which have a common structural hair-pin fold. Full length PYY_1−36_ is processed by DPP4 to PYY_3−36_ which then selectively binds to Y2 neuropeptide Y receptors. PYY is secreted post-prandially and acts as a satiety signal (36). Administration of PYY by infusion in human volunteers leads to reduced food intake and this effect is retained in patients with obesity (37, 38). PYY has a short half-life, limiting its therapeutic practicability. Specific alterations to the PYY_3−36_ sequence can be made to obtain an analog with a prolonged duration of action. Long-acting analogs of PYY which enable weekly injections are being tested in Phase I (ClinicalTrials.gov Identifier NCT01515319). Another analog, with an alpha-helix stabilizing sequence and histidine residues, demonstrated efficacy in reducing food intake for up to 24 h in animal studies (39). Reversible PEGylation of PYY(3–36) maintains its functional activity and significantly prolongs its half-life *in vivo* (40). An oral preparation of PYY_3−36_ has been tested and does reduce food intake during an *ad libitum* test meal, but did not affect total 24 h food intake after a single dose, suggesting that the effect of a single dose is short-lived (41).

Pancreatic polypeptide (PP) is a 36-amino acid peptide hormone released by the PP cells of the pancreas after a nutrient stimulus. It is secreted in proportion to the calories ingested and remains elevated for up to 6 h (42). Intraperitoneal injections of PP reduce food intake over a 4 h period in both lean and obese mice (43). A short 90-min infusion of PP significantly reduced food intake in healthy human volunteers, and this effect seems to last for 24 h, reducing cumulative food intake by a mean of 25% (44). A stable analog of pancreatic polypeptide, PP 1420, has undergone Phase I trials to confirm the tolerability of single ascending subcutaneous doses of the peptide, PP 1420, in healthy subjects (45). The 7TM Pharma PP analog did not lower body weight in Phase II trials and development of the drug appears to have been terminated.

## Amylin and its synergistic effects

Amylin is a peptide hormone co-secreted with insulin by the pancreatic beta cells in response to nutrient stimulus. It is a 37-amino acid peptide, derived from an 89 amino-acid precursor protein, referred to as preProIAPP. PreProIAPP is cleaved at the N-terminus resulting in ProIAPP and which is subsequently processed by PC-2. It has various roles including inhibiting glucagon secretion, delaying gastric emptying and acting as a satiety signal. Initial *in vivo* studies showed that intrahypothalamic injection of rat amylin dose dependently reduced feeding in rats for up to 8 h (46). There is also some evidence that amylin is capable of influencing food reward pathways (47). Pramlintide, an analog of Amylin, has been modified to prevent the formation of fibrils preserving stability and solubility of the peptide and has been approved by the FDA for diabetes treatment but not specifically for obesity. In a double-blind placebo-controlled study over 4 months, patients lost approximately 3 kg on average (48). Interestingly, amylin appears to have a synergistic effect with leptin: pre-treatment with amylin reduces the leptin resistance observed in obesity and leads to a mean weight loss of 12.7% when given in combination with recombinant leptin in a proof of concept trial (49, 50). Newer amylin analogs such as davalintide, PEGylated amylin and dual amylin/calcitonin agonists are in pre-clinical development for diabetes/obesity treatment, as yet there are no published clinical trials (47).

## Inhibiting ghrelin to reduce appetite

Ghrelin, a 28-amino acid peptide derived from preproghrelin, is secreted by the X/A like cells of the stomach and to a lesser extent the small intestine (51, 52). Through the action of ghrelin-O-transferase (GOAT), the peptide undergoes posttranslational acylation at the serine-3 residue linking it to octanoic acid (53). This modification allows the peptide to cross the blood-brain barrier and bind to its cognate receptor, the growth hormone secretagogue (GHS) receptor. Ghrelin levels rise to a maximum when fasting and fall after feeding (54). Ghrelin, unlike the other gut hormones, increases food intake when given to human volunteers (55), i.e., it is a “hunger hormone” that drives appetite. Consequently, there has been considerable interest in targeting the GHS receptor or inhibiting GOAT as an anti-obesity therapy, for example, utilizing antagonists, inverse agonists, ghrelin vaccines and GOAT enzyme inhibitors (56, 57). There has been varied success. For example, an *in vivo* study using a small molecule ghrelin receptor antagonist in diet-induced obese mice led to reduced food intake and weight loss of up to 15% (58). The ligand-independent constitutive activity of GHS receptor can be inhibited by inverse agonists to the receptor and one such drug has been trialed in Phase I. Interestingly this trial reports that the most common side effect is that of somnolence as well as a positive chronotropic effect on heart rate; these may limit the tolerability and long-term safety of the treatment, however this may open up the possibility that the drug is useful for insomnia (59).

## Oxyntomodulin and the dual GLP-1/glucagon agonist concept

Due to the limitations of using single gut hormones (for example GLP-1 alone), researchers have investigated the idea of using the synergism between certain gut hormones to obtain benefits in metabolism and weight regulation. The most well-developed example of this is the dual agonism of GLP-1 and glucagon. Glucagon is secreted by alpha-cells of the pancreatic islets after PC-2 processing of proglucagon (60). In contrast to GLP-1, glucagon's classical effects are to increase hepatic glucose output by stimulating gluconeogenesis and glycogenolysis (61), although knockout of the glucagon receptor in mice only modestly reduces glucose levels by 1–2 mmol/l (62). Glucagon inhibits food intake when given to humans (63).

The interest in glucagon as a partner for GLP-1 comes from three other observations. Glucagon increases energy expenditure, unlike GLP-1, and this effect is retained when the two are co-infused (64, 65). Although animal studies suggest that this thermogenesis is via the activation of brown adipose tissue (BAT), studies in human subjects show that glucagon does not increase BAT activation as assessed by infrared thermometry nor ^18^F-FDG uptake (66). This BAT-independent effect of glucagon on energy expenditure may be due to substrate cycling (67), however direct evidence for this effect in humans is currently lacking. The second observation is that the acute hyperglycaemia from glucagon can be counter-balanced by GLP-1 co-administration (64). The third observation is that the co-infusion of GLP-1 and glucagon leads to a synergistic suppression of food intake (65). Therefore, dual agonism of glucagon and GLP-1 receptors promises to deliver weight loss efficacy beyond that seen with GLP-1 alone, via synergistic anorectic effects, increased energy expenditure and without any hyperglycaemia arising from the glucagon component.

The prototypical dual GLP-1/glucagon agonist is oxyntomodulin (OXM), which is a peptide hormone secreted by the neuroendocrine L-cells of the intestine. Like glucagon and GLP-1, it is a product of preproglucagon post translational processing by PC1 in the gut. It was first discovered following early experiments showing that intestinal extracts contained an activity similar to pancreatic glucagon. An isolated fraction with glucagon properties was termed “enteroglucagon,” later found to include two peptides containing the entire glucagon sequence, glicentin and OXM (68). In addition to containing the glucagon sequence, OXM has an additional C-terminal 8-amino acid octapeptide (labeled IP-1 in Figure 3) (69). *In vitro* experiments show that OXM is a full agonist at both the GLP-1 and glucagon receptors, albeit with reduced binding affinities of 10- and 100-fold respectively compared to the cognate peptides (70, 71). Consistent with these receptor binding actions, research has shown that OXM potentiates glucose stimulated insulin secretion via the GLP-1 receptor and is an incretin in its own right (72, 73). Dakin and colleagues showed that ICV injection of OXM into rats inhibits feeding. Importantly, comparison with a “pair-fed group” given the mean food intake of the OXM-treated group showed that OXM treatment led to enhanced weight loss relative to the pair-fed group, implying that the peptide leads to an increase in energy expenditure (74). GLP-1 receptor activity mediates the anorectic action of OXM, but the additional weight loss effect requires glucagon receptor activity (71, 75–77). Thus, OXM represents a dual agonist at the GLP-1 and glucagon receptors, with each receptor respectively mediating anorectic and energy expenditure effects.

In a double-blind, placebo-controlled, cross over study, Cohen and colleagues showed that an intravenous infusion of OXM at 3 pmol/kg/min reduced mean food intake by 19% in healthy human volunteers (78). Wynne and colleagues carried out a double-blind, placebo-controlled, parallel group study, showing preprandial subcutaneous injection of 400 nmol OXM in healthy overweight or obese volunteers for 4 weeks resulted in a mean reduction of body weight by 2.4% (79). This was associated with a 19% reduction in energy intake. OXM, when given as subcutaneous injections for 4 days, does not alter resting energy expenditure, but increased activity-related energy expenditure and total energy expenditure (80).

Due to the intrinsic dual receptor activity of OXM, analogs can therefore be used as unimolecular GLP-1/glucagon dual agonists (81, 82). Structural modifications to OXM to stabilize it have been developed including PEGylation (83), fatty-acid acylation (84, 85), amino acid substitution and other modifications (84). Furthermore, structure-activity studies have identified analogs with superior potency compared to the native peptide (84). Day and colleagues described a PEGylated co-agonist, which when administered once a week, normalized adiposity and glucose tolerance in diet-induced obese mice (86). Another PEGylated long acting dual GLP-1/glucagon receptor agonist was tested *in vivo* in diet-induced obese mice and was shown to improve glycaemia, reduce food intake and induce substantial weight loss (87). Given the positive findings from these studies, several clinical trials of GLP-1/glucagon co-agonists are being carried out by a variety of pharmaceutical companies (82). One of the earliest candidates in this class, MEDI0382, has recently reported Phase I/II results with promising efficacy in improving glycaemia and reducing weight in obese diabetic patients when given for up to 41 days (88).

## Other dual/triple agonist combinations (PYY/GLP-1, PYY/OXM, GLP-1/GIP, GLP-1/GIP/glucagon)

Other combinations of analogs with putative synergistic effects on reduction in food intake have been explored. PYY(3-36) has been shown to possess synergistic anorectic effects when combined with GLP-1 or OXM in infusion studies (89, 90). Beglinger's group also showed that an oral combination of PYY/GLP-1 can reduce food intake during an *ad libitum* test meal to a greater extent than either of the two hormones alone (41). However, not all gut hormone combinations give additive/synergistic effects on food intake, for example PYY(3-36) and PP (91).

Another interesting “dual agonist” concept that is being explored is the glucose-dependent insulinotropic polypeptide GIP/GLP-1 co-agonist. GIP is an insulinotropic peptide released by the K-cells of the small intestine in response to nutrient ingestion (18). Therefore, GIP/GLP-1 co-agonists were explored as a means of enhancing the incretin effect. Finan and co-workers engineered peptides with balanced activity at the GLP-1 and GIP receptors, and these were modified with fatty-acid acylation or PEGylation to improve their pharmacokinetics. These GIP/GLP-1 dual agonists produced enhanced weight loss and improvements in glucose levels compared to GLP-1 agonists alone in animal studies. In a Phase I study in diabetic volunteers, they showed that one of the dual agonists can reduce HbA1c with minimal gastrointestinal side effects (92). This dual agonist (NNC0090-2746) went on to Phase II studies in obese diabetic patients over 12 weeks where it was shown to reduce HbA1c by 0.96% compared to placebo and to reduce weight by up to 1.8% relative to placebo, albeit its efficacy was not demonstrably superior to liraglutide (93). Extending this work on dual agonism, Finan and colleagues have also devised triple agonists of the GLP-1, GIP and glucagon receptors. In pre-clinical models, these new agents demonstrate better weight loss than the GLP-1/GIP dual agonist mentioned above (94). Clinical study results are awaited with the GLP-1/GIP/glucagon triple agonists.

## Triple agonism with GLP-1/OXM/PYY—the pathway to the “medical bypass”?

In our continuing efforts to devise a better therapy for obesity, we have taken a tack which is based on the known effects of bariatric surgery on gut hormone physiology. Patients who have undergone RYGB have much higher post-prandial levels of PYY, GLP-1, and OXM (95–98), secretion being triggered by direct and early exposure of jejeunal L-cells to nutrients through the bypass. It is postulated that the release of these hormones leads to a synergistic effect on food intake (via all three hormones) and increased insulin secretion (via GLP-1 and OXM). The latter process could account for the rapid improvements in glycaemia which are observed early on after surgery, although other factors such as the very low-calorie liquid diet imposed after surgery are also likely to contribute. To test the hypothesis that the elevation of these three hormones were responsible for the benefits of RYGB directly, our group devised a study in which a triple infusion of GLP-1, OXM, and PYY (the “GOP infusion”) was given to recreate the post-prandial levels observed in RYGB patients. This led to a significant mean reduction in food intake by 32% when given for 10 h or so to healthy volunteers even though the individual doses of each hormone were calibrated to be sub-anorectic (98). No significant issues with nausea were observed. Continuing studies are underway to examine the effects of GOP infusion when this is given for up to 28 days to obese diabetic patients. Regardless, the GOP infusion study suggests that triple hormone/agonist therapy is feasible and capable of synergistically reducing food intake to achieve better weight loss than single or even dual-agonism, perhaps recapitulating the effect of the RYGB without surgery: the “medical bypass.”

## Conclusions

GLP-1 therapy for obesity represents the first generation of gut hormone-based therapies for obesity. In the search for better efficacy than presently available, it seems that approaches targeting multiple satiety and metabolic pathways for the optimal effects on food intake and metabolism will be required, and there is much pharmaceutical development in this sector at present. It is hoped that these efforts will pay off in the form of an efficacious, well-tolerated, safe and even beneficial tool for fighting the global pandemic of obesity.

## Author contributions

Review written by DH and edited by TT and SB.

### Conflict of interest statement

The authors declare that the research was conducted in the absence of any commercial or financial relationships that could be construed as a potential conflict of interest.
